# Integrative Epigenomic and Targeted Protein Analysis in MRONJ: Correlating DNA Methylation with Bone Biomarkers

**DOI:** 10.3390/ijms262211208

**Published:** 2025-11-20

**Authors:** Raed Awadh Alshammari, Marwa Tantawy, Danxin Wang, Elysse Castro-Hall, Maria Abreu, Alessandro Villa, Joseph Katz, Lexie Shannon Holliday, Yan Gong

**Affiliations:** 1 Department of Pharmacotherapy and Translational Research and Center for Pharmacogenomics and Precision Medicine, College of Pharmacy, University of Florida, Gainesville, FL 32610, USA or rae.alshammari@uoh.edu.sa (R.A.A.); mtantawy@cop.ufl.edu (M.T.); danxin.wang@cop.ufl.edu (D.W.); 2 Department of Clinical Pharmacy, College of Pharmacy, University of Ha’il, Ha’il 55473, Saudi Arabia; 3 Miami Cancer Institute, Baptist Health South Florida, Miami, FL 33176, USA; elyssec@baptisthealth.net (E.C.-H.); maria.abreu@baptisthealth.net (M.A.); alessandro.villa@baptisthealth.net (A.V.); 4 Department of Oral Medicine, College of Dentistry, University of Florida, Gainesville, FL 32610, USA; jkatz@dental.ufl.edu; 5 Department of Orthodontics, College of Dentistry, University of Florida, Gainesville, FL 32610, USA; sholliday@dental.ufl.edu; 6 Cardio-Oncology Working Group, University of Florida Health Cancer Institute, Gainesville, FL 32610, USA

**Keywords:** medication-related osteonecrosis of the jaw (MRONJ), epigenetics, DNA methylation, epigenome-wide association study (EWAS), bone proteins biomarkers, bone remodeling

## Abstract

Medication-related osteonecrosis of the jaw (MRONJ) is a severe adverse effect of antiresorptive agents, including bisphosphonates (BPs) and denosumab (DMB). We conducted a case–control epigenome-wide association study (EWAS) of 24 cancer patients treated with BPs or BPs + DMB using the Infinium^®^ MethylationEPIC v2.0 to explore epigenetic differences associated with MRONJ. Differentially methylated positions (DMPs) and regions (DMRs) were assessed across three analyses: MRONJ vs. controls (main), BPs-MRONJ vs. BPs-controls, and BPs/DMB-MRONJ vs. BPs/DMB-controls. Eight plasma bone biomarkers were quantified by Luminex and correlated with top methylation sites. We identified 10 DMPs and 4 DMRs at suggestive significance (*p* < 1 × 10^−5^). cg1913766 in the *NOP56* promoter was hypomethylated in the main analysis (*p* = 2.19 × 10^−7^) and in BPs-MRONJ (*p* = 4.80 × 10^−6^), correlating with osteocalcin (*p* = 0.02 and 0.03, respectively). *TNXB* cg21289669 was hypermethylated in the main analyses (*p* = 6.31 × 10^−6^), and TNXB locus formed a DMR (*p* = 3.30 × 10^−10^) in the main and BPs-MRONJ analyses (*p* = 2.76 × 10^−7^). cg11392877 in *PDE8A* was hypomethylated in BPs/DMB-MRONJ (*p* = 5.35 × 10^−7^). *TRIM15* was a significant DMR in BPs-MRONJ and the main analysis (*p* = 3.30 × 10^−10^). *TRIM15*, *TNXB*, and *PDE8A* regulate collagen I, while *NOP56* supports ribosome biogenesis, potentially contributing to MRONJ. Given the small sample size, these findings are preliminary and validation in larger studies is warranted.

## 1. Introduction

Medication-related osteonecrosis of the jaw (MRONJ) is a rare adverse drug reaction (ADR) associated with the use of antiresorptive medications such as bisphosphonates (BPs) and denosumab (DMB) that is characterized by an exposed unhealed jawbone that does not heal within eight weeks [1]. The incidence of MRONJ in patients with cancer is 2.4% with DMB and 1.7% with Zoledronic acid [2]. The common underlying mechanisms of MRONJ are believed to be inhibition of bone remodeling and angiogenesis, with proposed contributing factors including inflammation, infection, or immune dysfunction [1].

Numerous pharmacogenomic studies, including genome-wide association studies (GWAS) and candidate gene studies, have been conducted to identify genetic variants associated with MRONJ and to understand the pathophysiology of MRONJ. The majority of variants discovered in these studies are located in non-coding regions [3,4,5], consistent with the broader GWAS literature in which ~90% of associations map to non-coding regions [6]. However, germline variation accounts for only the inherited part of MRONJ risk, whereas epigenetic, particularly DNA methylation, may reflect treatment-related exposure. In bone, DNA methylation at the promoters of osteoprotegerin (OPG) and RANKL has been found to modulate osteoclastogenesis [7].

Epigenetics is the study of heritable changes in gene expression without altering the DNA sequence [8]. There are three main epigenetic mechanisms through which gene expression can be altered: histone modifications, non-coding RNA, and DNA methylation [8]. DNA methylation is a process catalyzed by DNA methyltransferases (Dnmts) enzymes which add a methyl group to 5-cytosine rings that follow guanine. It serves as a key epigenetic mechanism that can either silence or activate gene expression, without altering the DNA sequence. Therefore, epigenome-wide association study (EWAS) could provide an additional layer of insight into the mechanism of MRONJ.

In this study, we conducted a case–control EWAS to identify DNA methylation differences between MRONJ cases and controls by investigating differentially methylated positions (DMPs) and regions (DMRs) in cancer patients treated with BPs and sequential BPs/DMB therapy. Additionally, we performed targeted protein analysis in the plasma samples of the same patients to quantify the level of several bone protein biomarkers, including osteocalcin (OC), osteoprotegerin (OPG), osteopontin (OPN), sclerostin (SOST), interleukin-6 (IL-6), interleukin 1β (IL-1B), tumor necrosis factor α (TNF-α), and receptor activator of nuclear factor kappa B-ligand (RANKL). We then evaluated the correlation of these protein markers with the top DMPs.

EWAS approaches have been applied in bone-related conditions such as osteoporosis and osteoarthritis. However, to the best of our knowledge, there are no existing studies that examine EWAS aspects of MRONJ, which is a major gap in our understanding of the condition. By integrating targeted protein analysis with DNA methylation, we aim to fill this knowledge gap by studying differential DNA methylations that affect bone biomarker expression, thereby improving our understanding of the molecular mechanisms of MRONJ.

## 2. Results

In this study, we included 24 patients with cancer who were treated with either BPs or DMB. Among these, 12 were MRONJ cases and 12 were controls. Among the 15 patients treated with BPs, seven had MRONJ and eight were controls. Among the nine patients treated with BPs and DMB sequentially, five had MRONJ, and four were controls.

There were no significant differences in the demographic characteristics, such as age, sex, BMI, race, or smoking status, between MRONJ and controls (Table 1). In the MRONJ group, 58.3% of patients were White, and 33.3% were Black, while the control group included 83.3% White and 8.3% Black individuals. Among the 12 MRONJ cases, 58.3% were in stage 1 and 25% in stage 2 based on the American Association of Oral and Maxillofacial Surgeons (AAOMS) staging system, with stages 3 and 4 unrepresented. Staging information was not available for 2 patients (16.7%). Lesion locations were predominantly in the mandible (58.3%), with 16.7% in the maxilla, and 8.3% in the posterior. Lesion locations were not documented for 2 patients (16.7%). Bassline characteristics of participants for the subgroup analyses are provided in the Supplementary Data (Tables S1 and S2).

**Figure 1:**
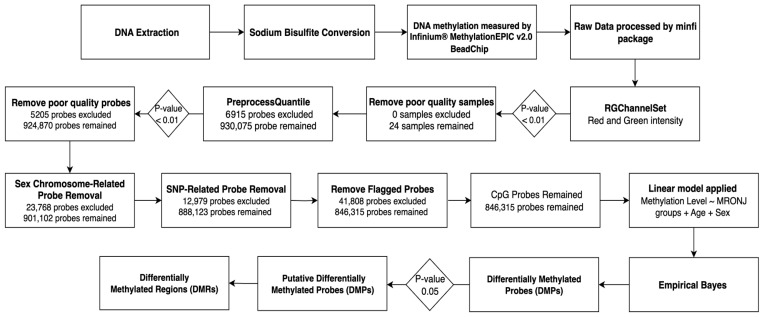
Flowchart of EWAS quality control steps and analyses.

The EWAS quality control (QC) steps and analysis workflow are summarized in Figure 1. After the QC steps, a total of 846,315 CpG probes remained for differential methylation analyses. As shown in the method, we performed three analyses in this study. The main analysis (MRONJ vs. controls) and two subgroup analyses: first, (BPs-MRONJ vs. BPs controls), and second, (BPs/DMB-MRONJ vs. BPs/DMB controls) were performed. DMPs and DMRs were analyzed for all three analyses.

### 2.1. Differentially Methylated Probes

#### 2.1.1. Main Analysis: MRONJ vs. Controls

**Figure 2:**
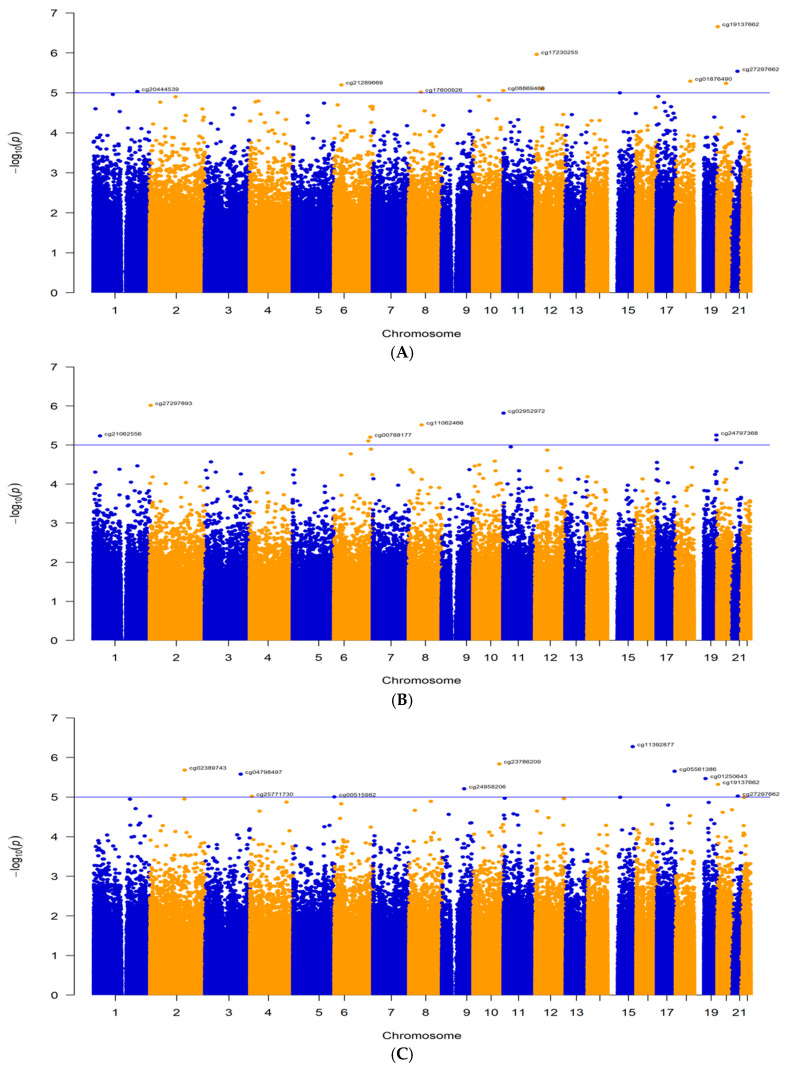
Manhattan plots for the three EWAS of MRONJ: (**A**). the main analysis (λ = 1.45); (**B**). the first subgroup analysis (λ = 1.09); (**C**). the second subgroup analysis (λ = 1.16). The x-axis represents the chromosome position, and the y-axis represents the −log10(p) value. Each blue or yellow dot represents a CpG site across chromosomes.

**Figure 3:**
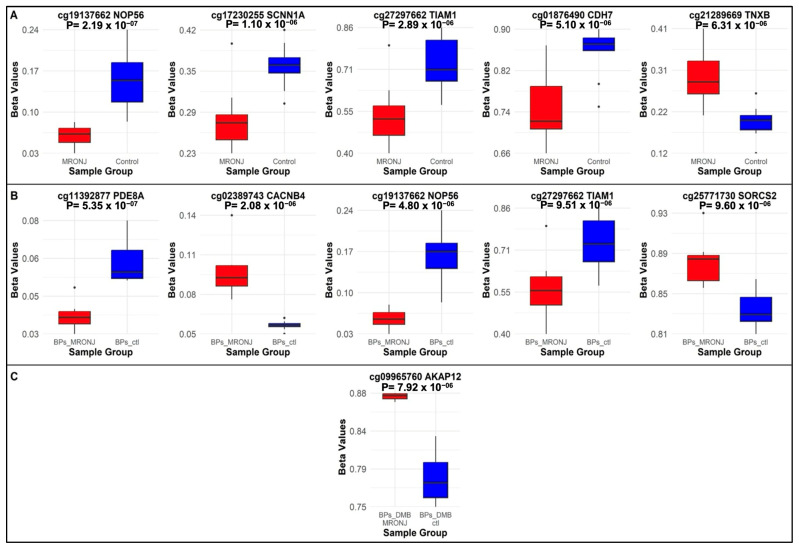
Boxplots showing the methylation differences in the beta values of the top DMPs: (**A**) in the main analysis; (**B**) in the first subgroup analysis; (**C**) in the second subgroup analysis.

In the main analysis, none of the CpG sites reached the Bonferroni-corrected epigenome-wide significant threshold of 5.91 × 10^−8^. However, ten DMPs met the suggestive level of significance (*p* < 1 × 10^−5^) (Table S3; Figure 2A). Two of these DMPs are located in promoter regions, four in intronic regions, two in exons, one intergenic, and one in a 3′-untranslated region (UTR). Among these DMPs, five are hypomethylated and five are hypermethylated (Table S3). Five DMPs had a plausible mechanistic explanation for how they might be involved in the development of MRONJ: cg19137662 in the *NOP56* promoter (*p* = 2.19 × 10^−7^, logFC = −1.38), cg17230255 in the *SCNN1A* promoter (*p* = 1.10 × 10^−6^, logFC = −0.60), cg27297662 in the *TIAM1* intron (*p* = 2.89 × 10^−6^, logFC = −1.30), cg01876490 in the *CDH7* intron (*p* = 5.10 × 10^−6^, logFC = −1.10), and cg21289669 in the *TNXB* exon (*p* = 6.31 × 10^−6^, logFC = 0.84) (Table 1). The beta values of these five DMPs in MRONJ vs. controls are shown in Figure 3A.

#### 2.1.2. First Subgroup Analysis: BPs-MRONJ vs. BPs-Controls

Eleven DMPs were associated with BPs MRONJ at the suggestive level of *p* < 1 × 10^−5^ in the first subgroup analysis, eight were located in promoter regions and three in introns (Figure 2B, Table S4). Among these DMPs, four were hypermethylated and six were hypomethylated. Five DMPs are within genes that have plausible mechanistic explanations for MRONJ. These DMPs include cg11392877 in the *PDE8A* promoter (*p* = 5.35 × 10^−7^, logFC = −0.98), cg02389743 in the *CACNB4* promoter (*p* = 2.08 × 10^−6^, logFC = 0.92), cg19137662 in the NOP56 promoter (*p* = 4.80 × 10^−6^, logFC = −1.61), cg27297662 in the *TIAM1* intron (*p* = 9.51 × 10^−6^, logFC = −1.53), and cg20000507 in the *RACK1* promoter (*p* = 9.94 × 10^−6^, logFC = −0.81) (Table 2). The beta values of these five DMPs in BPs-MRONJ and BPs-controls are illustrated in Figure 3B.

#### 2.1.3. Second Subgroup Analysis: BPs/DMB MRONJ vs. BPs/DMB

Eight DMPs were associated with BPs/DMB-MRONJ at the suggestive level of *p* < 1 × 10^−5^ (Table S5). One DMP that is hypermethylated and has a possible mechanistic explanation is located in the intronic region of *AKAP12* (*p* = 7.92 × 10^−6^, logFC = 0.89) (Figure 2C). The beta values of these five DMPs in BPs/DMB-MRONJ and BPs/DMB-controls are illustrated in Figure 3B.

### 2.2. DMRs

Four DMRs were identified at the suggestive level of *p* < 1 × 10^−5^ in the DMRs analysis and have plausible mechanistic explanations in MRONJ. Three of four DMRs were identified in the main analysis. Two DMRs were statistically significant, one DMR in the main analysis and another one in the first subgroup analysis. (Table 3 and Tables S6–S8).

The top DMR in the main analysis was *TNXB,* which included 20 DMPs (*p* = 3.30 × 10^−10^, mean methylation difference = 0.07). This DMR was associated with MRONJ in the first subgroup analysis of BPs MRONJ vs. BPs controls, with 10 DMPs found within the *TNXB* gene (*p* = 2.76 × 10^−7^, mean methylation difference = 0.09) (Table 3).

*SPON2* was identified as a DMR in both the main analysis and the first subgroup analysis. In the main analysis, the DMR in *SPON2* included 13 CpGs (*p* = 2.76 × 10^−7^, mean methylation difference = 0.03). In the first subgroup analysis, the DMR comprised 12 CpGs within *SPON2* (*p* = 4.27 × 10^−8^, mean methylation difference = 0.04) were identified.

*TRIM15* was significant in the first subgroup analysis, comprising 12 CpGs within *TRIM15* (*p* = 3.30 × 10^−10^, mean methylation difference = 0.10), while this DMR was also associated with MRONJ in the main analysis with 9 CpGs (*p* = 4.13 × 10^−5^, mean methylation difference = 0.10) (Figures S1 and S2).

In addition, one DMR was unique and was marginally significant in the main analysis. It involved a group of Homeobox genes, *HOXB-AS3*, *HOXB3*, and *HOXB4*, including 15 CpGs (*p* = 4.27 × 10^−8^, mean methylation difference = 0.07).

### 2.3. Bone Protein Biomarkers

**Figure 4:**
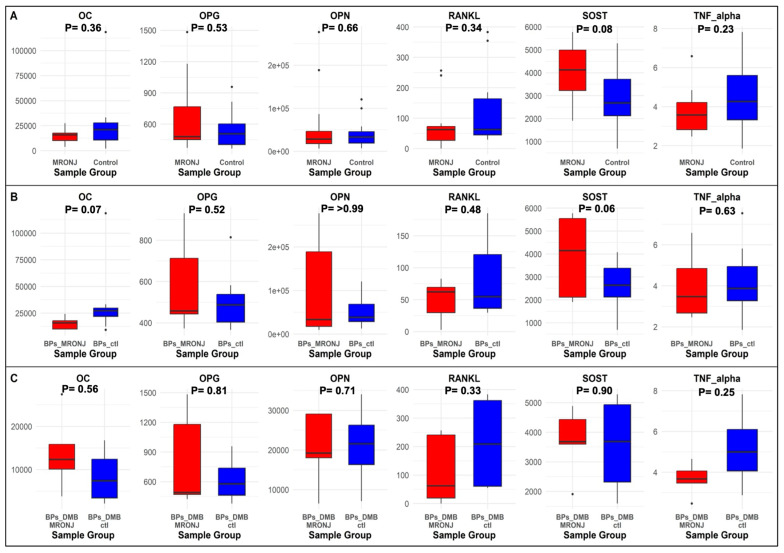
Boxplots of bone protein biomarker levels: (**A**). in the main analysis; (**B**). in the first subgroup analysis; (**C**). in the second subgroup analysis.

We also measured the protein levels of bone biomarkers OC, OPG, OPN, SOST, TNF-α, RANKL, IL-6, and IL-1B in these 24 patients. Six biomarkers passed quality control: OC, OPG, OPN, SOST, TNF-α, and RANKL (Table S9). The levels of these six protein biomarkers in MRONJ vs. controls are presented in Figure 4. In the main and subgroup analyses, none of the biomarkers had a significant difference between MRONJ and controls. Notably, the concentration of SOST was marginally higher in the MRONJ patients compared to controls in the main analysis (*p* = 0.08) and in the first subgroup analysis (*p* = 0.06) (Figure 4). The biomarker OC had a trend to be lower in the BP MRONJ patients compared to the BP controls in the first subgroup analysis (*p* = 0.07). However, the trend was in the opposite direction: higher in BPs/DMB MRONJ compared to BPs/DMB controls, among the patients treated with BPs/DMB sequentially. The *p*-value was 0.36 in the combined analysis of MRONJ vs. controls.

### 2.4. Correlation Analysis

To gain a more comprehensive understanding of the molecular mechanism of MRONJ, we assessed the correlation between DNA methylation and the bone protein biomarker levels in these patients. Pearson correlation analysis was performed to assess the relationship between bone biomarkers and significant DMPs in both the main analysis and subgroup analyses. In the main analysis, OC showed a significant moderate positive correlation with cg19137662, which is located in the promoter region of *NOP56* (r = 0.48, *p* = 0.02) (Table 4; Figures S3 and S4). In the first subgroup analysis, the correlation coefficient was higher, indicating a stronger positive relationship (r = 0.57); however, the statistical significance was slightly reduced (*p* = 0.03) (Figures S5 and S6). The same DMP demonstrated a significant moderate positive correlation with RANKL in the second subgroup analysis (r = 0.73, *p* = 0.03) (Figures S7 and S8). Additionally, TNF-α showed a significant moderate correlation with cg17230255 in the promoter region of *SCNN1A* in both the main analysis and the first subgroup analysis (r = 0.56, *p* = 0.004; r = 0.66, *p* = 0.01). In contrast, SOST showed a borderline significant negative correlation with cg11392877 in the promoter region of *PDE8A* in the first subgroup analysis (r = −0.51, *p* = 0.05). The same DMP had a significantly positive correlation with OPG in the second subgroup analysis (r = 0.82, *p* = 0.01). However, in the second subgroup analysis, the correlation coefficient was not as strong (r = 0.63, *p* = 0.07). No other correlations tested reached statistical significance (*p* ≥ 0.05).

## 3. Discussion

In this first EWAS analysis of MRONJ, we evaluated DNA methylations in MRONJ cases vs. controls in cancer patients treated with BP or DMB. We also evaluated cases of MRONJ induced by BPs alone or sequential BPs/DMB treatments, comparing them with patients with the same treatments but who had not developed MRONJ. We identified a total of ten DMPs and four DMRs that provide plausible mechanistic explanations for the development of MRONJ. A few of these DMPs also correlated with bone protein marker levels in plasma.

The direct action of BPs is thought to be primarily or exclusively on osteoclasts [9]. DMB directly inhibits osteoclast differentiation and also has direct effects on immune cells [10]. Many of the genes that we found associated with DMPs and DMRs have previously been identified as being expressed in osteoclasts, and some are known regulators of osteoclast activity. The genes differentially regulated by methylation fall into several functional categories: regulators of ribosome formation, ion channels, cytoskeletal regulators, extracellular matrix components, cyclic AMP-phosphodiesterase, vesicle sorting apparatus proteins, signal transduction scaffold proteins, E3-ubiquitin ligases, and homeobox genes, including long non-coding RNAs.

The top hit DMP was cg19137662 in the promoter of the *NOP56* gene, which encodes the NOP56 ribonuclear protein. cg19137662 is located in regions enriched for H3K4me3 and H3K27ac histone marks, which are typically associated with active promoter regions. This CpG also shows strong DNase I hypersensitivity signal intensity, indicating open chromatin accessibility, and is located near a defined transcription start site (TSS) peak, supporting its potential regulatory function [11]. NOP56 is a nucleolar protein involved in ribosome formation. It is unclear how this gene might be associated with MRONJ, but it is linked to cancer and the neurodegenerative disease, spinocerebellar ataxia type 36 [12]. In our study, we found an intriguing correlation of this CpG with plasma OC levels, which is a marker for bone turnover. In BPs-treated patients, a moderately significant positive correlation between methylation of cg19137662 and OC levels was detected in both MRONJ and controls. In patients who were treated with BPs, then shifted to DMB, higher methylation of cg19137662 significantly correlated with lower OC levels. This suggests that a fundamental change in bone turnover occurred that may be related to the increased rate of MRONJ in patients who switch from BPs to DMBs.

Two different DMPs associated with ion channels were identified. Cg17230255 is located in the promoter region of *SCNN1A*, a subunit of the epithelial sodium channel (ENaC). This DMP is in a region enriched with H3K4me3 and H3K27ac histone marks and overlaps with DNase I hypersensitivity peak, indicating open chromatin and active promoter-associated regulatory potential [11]. ENaC is expressed in osteoclasts and may have a regulatory function [13,14]. Interestingly, a study reported that BPs stimulate non-selective cation channels, including ENaC, and it was postulated that this might contribute to their ability to inhibit osteoclasts [15]. Methylation of the cg17230255 was moderately correlated with TNFα plasma levels, and GeneMANIA network analysis shows a link between *SCNN1A* and TNFα. The second channel identified was the voltage-gated calcium channel subunit gene *CACNB4*, which harbors DMP cg02389743 located in a CpG island and the *CACNB4* promoter. This site overlaps with regions enriched for H3K4me3 and H3K27ac histone marks, as well as a DNase I hypersensitivity peak, indicating an active promoter and open chromatin configuration [11]. The *CACNB4* gene codes for calcium voltage-gated channel auxiliary subunit beta 4, part of a voltage-dependent calcium channel complex that mediates calcium influx into cells upon membrane polarization. It is linked to neurological disorders, including episodic ataxia and epilepsy. In osteoclasts, calcium signaling plays a vital role, and voltage-gated calcium channels have been implicated [16]. The *CACNB4* gene inhibits wnt/beta catenin signaling, which is a central regulatory pathway in bone formation by osteoblasts [17,18]. Changes in *CACNB4* expression due to differential methylation could alter the bone response to BPs or DMB.

Osteoclasts depend on a complex cytoskeletal structure called the actin ring for bone resorption [19]. BPs disrupt the prenylation of rho-GTPases, vital regulators of actin ring formation [9]. Cg27297662 is an intron in the *TIAM1*, which encodes T-cell lymphoma invasion and metastasis-inducing protein 1 (TIAM1), a guanine exchange factor (GEF) that activates various rho-class GTPases [20]. This DMP lies within an enhancer-like structure enriched with H3K4me1 and weak H3K27ac signals and overlaps a DNase I hypersensitivity peak [11]. TIAM1 has been shown to be a crucial regulator of podosomes/invadapodia, which are discrete, dynamic, actin structures that in osteoclasts are organized to form actin rings [21,22]. Although *TIAM1* has not been studied in osteoclasts, it is known to be associated with cortactin in other cell types, and cortactin is a crucial protein controlling the actin ring of osteoclasts [23,24,25].

*TNXB* has been identified as both a DMP and a DMR. Cg21289669 is in a CpG island in an exon of *TNXB* gene that encodes tenascin X. This DMP is located in a region characterized by enhancer-associated histone marks (H3K4me1 and H3K27ac) and DNase I hypersensitivity, consistent with open chromatin and active regulatory function [11]. This large extracellular matrix protein regulates the deposition of collagen and provides structural support for elastic fibers. Mutations result in Ehlers-Danlos syndrome, which includes bone loss as part of the pathology. This is due to increased osteoclast activity without affecting osteoblast bone formation [26]. Changes in expression of tenascin X due to differential methylation would be expected to alter bone remodeling by affecting the extracellular matrix.

Cyclic AMP is an important second messenger regulating bone remodeling [27]. The DMP cg11392877 is in the promoter of phosphodiesterase 8A (*PDE8A*), an enzyme that breaks down cAMP. Chromatin annotation indicates that this DMP lies within an enhancer–promoter region enriched with H3K4me1, H3K4me3, and H3K27ac histone marks and exhibits an elevated DNase I hypersensitivity signal, suggesting open and transcriptionally active chromatin [11]. In osteoclasts, cAMP inhibits resorptive activity [28] while in osteoblasts, cAMP promotes bone formation [29]. Knockdown of phosphodiesterase 8A by RNA interference inhibited the differentiation and activity of osteoblasts in vitro, providing evidence that it is involved in regulating bone remodeling [30]. Alterations in phosphodiesterase 8A expression would be expected to alter bone remodeling.

*AKAP12* and *RACK1* encode proteins that serve as scaffolding proteins in signal transduction pathways. A–kinase–anchoring protein-12 serves to compartmentalize components of the cyclic AMP-signaling pathway to sites in the plasma membrane [31]. It has been linked to the regulation of osteoclasts and osteoblasts and exacerbates rheumatoid arthritis [32]. Receptor for Activated Protein Kinase C 1 anchors protein kinase C, c-Src, and other signaling molecules to ribosomes and to focal adhesions at the plasma membrane [33,34]. Its interaction with c-Src was reported to be crucial for the organization of osteoclasts’ actin rings and bone resorption [35]. cg09965760 is found in an intron of *AKAP12*. *cg00515982* is present in the promoter of *RACK1*.

A DMR was identified in *TRIM15*. This gene codes tripartite motif-containing protein 15, an E3 ubiquitin ligase that is involved in the regulation involving AKT/FOX01/LASP, which has been linked to oncogenesis [36]. Recent data from a study published in 2025 show that this regulation is involved in chondrocyte senescence and the progression of osteoarthritis [37].

A unique DMR was identified associated with *HOXB-AS3*, *HOXB3*, and *HOXB4*. These homeobox genes are part of a family that regulates organogenesis [38]. *HOXB3* is linked to angiogenesis [39]. In endothelial cells, HOXB3 drives ephrin-A1 expression and promotes sprout-to-tube formation; notably, the endothelial cells that are maturing into capillaries show HOXB3 expression [40]. During endothelial differentiation of human mesenchymal stem cells, HOXB3 levels rise sharply by day 21 [41]. Given that MRONJ involves impaired vascularization and wound repair in the jaw, methylation shifts across the HOXB-AS3/HOXB3/HOXB4 locus could plausibly influence the angiogenic responses.

Plasma levels of OC, OPG, OPN, SOST, TNF-α, and RANKL were measured but did not differ significantly between MRONJ and controls in the main and the subgroups analyses. However, SOST levels showed a trend toward being higher in MRONJ patients in both the main analysis and the BP subgroup. This observation is biologically plausible, as sclerostin is a Wnt pathway inhibitor secreted by osteocytes that reduces osteoblast activity and bone formation. BPs have been shown to upregulate SOST expression, thereby further suppressing Wnt signaling and contributing to decreased bone formation [42]. Thus, higher circulating levels may reflect suppressed bone remodeling in MRONJ. On the other hand, OC levels showed more variability. In the BPs-treated subgroup, OC tended to be lower in MRONJ patients compared with controls, which may suggest impaired bone formation activity under bisphosphonate treatment. In contrast, in the sequential BPs/DMB subgroup, the trend was reversed, with higher OC levels in MRONJ compared with controls. Future research should prioritize local biomarker sources such as saliva or gingival crevicular fluid and employ larger sample sizes to enhance sensitivity in identifying molecular alterations linked to MRONJ.

This study has some limitations. First, we had a small sample size, which makes it difficult to capture significant signals at the epigenome-wide level. Based on that, a relaxed *p*-value threshold was used in DMPs and DMRs, and a less conservative approach was used in EWAS. We utilized 1 × 10^−5^ as a suggestive threshold to define suggestive DMPs. The same approach was used for the DMRs by using a *p*-value < 0.05 to define putative DMPs to obtain them. Secondly, we lacked information on patients’ oral hygiene practices and did not incorporate cancer type into our analyses. Both factors may influence MRONJ risk and therefore represent potential sources of residual confounding. The third limitation is that the DNA samples were taken from whole blood. As DNA methylation is tissue-specific, methylation differences between cases and controls could occur due to different cell types within and between individuals. We used the EpiDISH package to estimate cell type proportions in order to include them as covariates in our analysis. However, due to the limitation of a small sample size, there was a risk of overfitting, so we did include it in the final model. We also aimed to assess whether there were significant differences in estimated cell type proportions between MRONJ cases and controls. No statistically significant differences were observed, which may be attributed to the limited sample size. Since EpiDISH relies on reference-based deconvolution, the small sample size may have affected the accuracy and stability of the cell type estimations. The results of this analysis are provided in the Supplementary File (Tables S10–S12; Figure S9).

As MRONJ is a localized condition affecting the jawbone, and our study relies on methylation profiles derived from whole blood, there is an inherent limitation in tissue specificity. While blood is a practical and accessible surrogate tissue, its use assumes that systemic methylation changes reflect local disease processes in the jaw. This assumption is difficult to validate and may not hold true in all cases. It is possible that some of the differential methylation signals observed, particularly those related to immune pathways or inflammatory markers, reflect systemic immune changes rather than localized bone pathology. Future studies should aim to replicate and validate these findings in jawbone tissue or more localized biological sources, such as saliva.

## 4. Materials and Methods

This study was a retrospective case–control study. The data consists of de-identified clinical records from the Miami Cancer Institute and the University of Florida (UF) Dental Clinic. This study included 24 cancer patients treated with intravenous BPs (zoledronate or pamidronate) (*n* = 15) or BPs and DMB sequentially (*n* = 9). The definitions of cases and controls are as follows: patients who were treated with BPs or DMB and developed MRONJ according to the clinical criteria set by the AAOMS [1]. Controls are patients treated with BPs or DMB for at least two years without developing MRONJ and were matched to the MRONJ cases in terms of the type of medications. This study was approved by the Institutional Review Boards at the University of Florida (Gainesville, FL, USA) and Miami Cancer Institute (Miami, FL, USA) in the USA. Written informed consent was obtained from all participants in the study.

### 4.1. DNA Methylation Profiling and Processing

Whole blood samples were collected after initiation of antiresorptive medication and prior to the clinical diagnosis of MRONJ. Samples were processed at the Center for Pharmacogenomics and Precision Medicine at the College of Pharmacy at UF. DNA was isolated and extracted from buffy coat using the QIAamp DNA Mini Kit. Following Illumina’s guidelines, samples underwent bisulfite treatment with a consistent DNA input of 500 ng per sample. The analysis for genome-wide DNA methylation utilized the Illumina Infinium MethylationEPIC v2.0 BeadChip (Illumina Inc., San Diego, CA, USA) and was performed at the Illumina service center at the University of Miami Hussman Institute for Human Genomics, adhering strictly to the manufacturer’s provided protocols.

#### 4.1.1. Data Processing and Quality Controls (QC)

The preprocessing of the original array data (IDAT files) as an RGChannel Set at the probe level (red and green) was carried out using R (version 4.3.3) and the Bioconductor (version 3.18) minfi package (version 1.48.0) [43] with the function read.metharray.exp to determine DNA methylation levels.

#### 4.1.2. Assessment of Samples Quality and Normalization

A mean detection *p*-value cutoff of >0.01 was used to exclude poor-quality samples. None of the samples were excluded. Additionally, quantile normalization was performed to address technical variability and make methylation level distributions across all samples consistent which improved the reliability and accuracy of downstream statistical analyses.

#### 4.1.3. Gender Confirmation

PlotSex in the minfi package was used to validate individual’s gender by checking the concordance between genetically estimated sex and self-reported gender [43].This function computed the median log2 of the total intensity value for each sex chromosome. It confirmed the accuracy of self-reported gender data.

#### 4.1.4. Removing Poor-Quality Probes

To exclude poor-quality probes, a *p*-value threshold of 0.01 was used, as signals below this threshold were considered unreliable and indistinguishable from background noise. A total of 5205 probes with *p*-value > 0.01 were considered poor quality and excluded from further analyses.

#### 4.1.5. Removing Probes at Sex Chromosomes and SNPs

In EWAS, it is a common practice to remove probes located on the sex chromosomes due to X chromosome inactivation, which can affect the results. Therefore, a total of 23,768 probes located on sex chromosomes were removed from the analysis. Additionally, probes located in SNPs were also excluded to avoid potential confounding effects. This was done using the basic function of the minfi package, dropLociWithSnps, which removed 12,979 probes. These steps were taken to enhance the accuracy and reliability of the methylation data analysis.

#### 4.1.6. Removing Flagged Probes

Illumina identifies a list of probes as flagged probes, also known as cross-reactive probes, which can bind to multiple loci. These cross-reactive probes can produce unreliable signals because they can hybridize to more than one location in the genome, leading to ambiguous or misleading results. We removed 41,808 probes for this reason.

After the QC steps, 846,315 CpG probes were included in the analysis. The final DNA methylation beta values and M values were calculated. Beta values represent the percentage of methylation at each site, while M values are the logit transformation of beta values. M values were used in all statistical analyses due to better statistical properties [44], while beta values were also reported to better interpret the results. With a sample size of 24 and α = 5.9 × 10^−8^, there is ≥80% power to detect a 10% methylation difference at approximately 38.6% of CpG sites, as estimated using EPIC Array Power Calculations [45].

### 4.2. Assessment of Cytokine and Bone Protein Levels

The levels of OC, OPG, OPN, SOST, IL-6, IL-1B, TNF-α and RANKL in the plasma samples of the 24 patients were determined using Milliplex multiplex assay HBNMAG-51K and singleplex assay HRNKLMAG-51K-01 (EMD, Millipore, Burlington, MA, USA). Plasma was normalized based on volume as recommended by the manufacturer’s instructions. Data were obtained on a MAGPIX multiplex reader system running xPONENT 3.1 software (Luminex, Austin, TX, USA) and examined using a five-parameter logistic spline-curve fitting method and Milliplex Analyst V5.1 software (Vigene Tech, Carlisle, MA, USA).

During the QC process, protein concentrations below the lower limit of detection were assigned a value of half the lower detection limit. For concentrations exceeding the upper limit of detection, the highest measurable value was divided by two. These adjustments were applied to ensure data consistency and accuracy in subsequent analyses. Two proteins, IL-6 and IL-1B, had 83.3% and 66.7% of samples, respectively, with concentrations below the lower limit of detection and were excluded from further analysis.

### 4.3. Statistical Analysis

#### 4.3.1. Descriptive Statistics

The patient characteristics were summarized by MRONJ status, including median, interquartile range (IQR for continuous variables, as well as frequency and percentage for categorical variables. To assess differences between MRONJ and controls, *t*-test was used for continuous variables that were normally distributed such as age, while the Wilcoxon rank-sum test was applied to variables that were not normally distributed such as BMI. For categorical variables such as sex and smoking status, Fisher’s exact test was utilized. *t*-test was used to assess the group difference in bone protein levels between MRONJ and controls in the three analyses.

#### 4.3.2. Estimating Cell Type Proportions

DNA methylation is tissue-specific, with different cell types exhibiting distinct methylation patterns. The Epigenetic Dissection of Intra-Sample Heterogeneity (EpiDISH) package (version 2.18.0) was used as a reference-based method, utilizing the centDHSbloodDMC.m implemented reference. This method calculated the proportions of B cells, natural killer (NK) cells, CD4+ T cells, CD8+ T cells, monocytes, neutrophils, and eosinophils using three methods: Robust Partial Correlations (RPC), Cibersort (CBS), and Constrained Projection (CP), also known as Houseman.

Our initial goal was to include the estimated cell proportions as covariates in the EWAS model to adjust for potential confounding. However, due to the small sample size and the resulting risk of overfitting, we did not include these adjustments in the final model. Instead, we compared estimated cell type proportions between MRONJ cases and controls using the Wilcoxon rank-sum test.

#### 4.3.3. DMPs Analysis

A multiple linear regression model was conducted using the limma package to identify DMPs. The M values for each CpG were used as the independent variable, with subject status (MRONJ or control) as the dependent variable, adjusting for age and sex as covariates. To account for multiple testing, we applied a Bonferroni correction across 846,315 CpG sites, resulting in a genome-wide significance threshold of *p* < 5.9 × 10^−8^. In addition, a cutoff of *p* < 1 × 10^−5^ was used as a suggestive threshold to identify potentially meaningful DMPs. DMPs were identified in the main analysis comparing MRONJ to controls. Given that some patients were treated with BPs or sequential therapy with BPs and DMB, two subgroup analyses based on prescribed medications were performed to further identify DMPs. The rationale for these analyses was two-fold: first, to determine whether a specific subgroup was driving the main analysis; second, to assess whether the identified DMPs were specific to certain medications or were associated with the main analysis independent of medications.

A DMP is assigned as a promoter feature if it is located within 2 KB upstream of the transcription start site (TSS) or is identified by the Encyclopedia of DNA Elements (ENCODE), Genome-wide integration of enhancers and target genes in GeneCards (GeneHancer) datasets or validated by the Eukaryotic Promoter Database, regardless of the genomic feature.

#### 4.3.4. DMRs Analyses

To calculate DMRs, we first identified DMPs with a *p*-value < 0.05 as putative DMPs in each analysis separately. These DMPs were then used as input for the DMRcate package (version 2.16.1) [46], which uses a Gaussian kernel smoothing to adjust squared EWAS t statistics at each CpG site, and the significant CpG sites within a certain distance are combined to form DMRs. Specifically, a bandwidth of 1000–2000 nucleotides was applied to ensure that CpG sites within proximity were considered part of the same region.

## 5. Conclusions

We identified several genes that are differentially methylated in MRONJ and controls, which may serve as potential biomarkers. These findings suggest that MRONJ may be epigenetically influenced. However, given the small sample size, the study is at increased risk of type II error, and the results should be considered preliminary. Further investigations in larger, independent cohorts are warranted to validate and clarify the implications of these associations.

## Figures and Tables

**Table 1:** Clinical characteristics of study participants.

Variable	All Participants (*n* = 24)		
	Cases (*n* = 12)	Control (*n* = 12)	*p* Value
* Continuous			
Age (years)	68.5 (59.25–71.5)	67.5 (57.0–72.0)	0.73
BMI (kg/m^2^)	28.4 (24.5–32.1)	27.25 (25.4–28.8)	0.71
* Categorical			
Sex at birth			>0.99
Female	8 (66.7.%)	9 (75%)	
Male	4 (33.3%)	3 (25%)	
Race Groups			0.32
White	8 (66.7%)	11 (91.7%)	
Black	4 (33.3%)	1 (8.3%)	
Smoking status			>0.99
Current smoker	0 (0.0%)	1 (8.3%)	
Previous smoker	7 (58.3%)	7 (58.3%)	
Never smoke	5 (41.7%)	4 (33.3%)	
AAOMS Staging of MRONJ			
Stage 1	7 (58.3%)	-	
Stage2	3 (25%)	-	
Stage3	0 (0.0%)	-	
Stage4	0 (0.0%)	-	
NA	2 (16.7%)		
Lesion location			
Maxilla	2 (16.7%)	-	
Mandible	7 (58.3%)	-	
Anterior–posterior	1 (8.3%)	-	
Lingual/buccal	0 (0.0%)	-	
NA	2 (16.7%)	-	
Medications			
Bisphosphonates	7 (58.3%)	8 (66.7%)	>0.99
Sequential therapy (BPs/DMB)	5 (41.7%)	4 (33.3%)	

Abbreviations: MRONJ: medication-related osteonecrosis of the jaw; BMI: body mass index; AAOMS: American Association of Oral and Maxillofacial Surgeons; NA: Not Available. * For continuous variables, median and (IQR) were reported; for categorical variables, numbers (%) were presented. Age was compared between groups using *t*-test, and BMI using Wilcoxon rank-sum test.

**Table 2:** Summary of DMPs associated with MRONJ in the main and subgroup analyses.

No	Ilumina ID	CHR	MAP INFO	Gene Name	Feature	Main Analysis		First Subgroup Analysis		Second Subgroup Analysis	
						logFC	*p*	logFC	*p*	logFC	*p*
1	cg19137662 *	chr20	2652716	*NOP56*	Promoter	−1.38	2.19 × 10^−7^	−1.60	4.80 × 10^−6^	−1.01	0.01
2	cg17230255 *	chr12	6364225	*SCNN1A*	Promoter	−0.60	1.10 × 10^−6^	−0.68	2.27 × 10^−5^	−0.45	0.01
3	cg27297662	chr21	31136999	*TIAM1*	Intron	−1.30	2.89 × 10^−6^	−1.53	9.51 × 10^−6^	−0.85	0.02
4	cg01876490	chr18	65766883	*CDH7*	Intron	−1.10	5.10 × 10^−6^	−1.17	0.0002	−0.97	0.01
5	cg21289669	chr6	32096987	*TNXB*	Exon	0.84	6.31 × 10^−6^	0.88	0.0003	0.82	0.005
6	cg11392877	chr15	84981545	*PDE8A*	Promoter	−0.55	0.0005	−0.98	5.35 × 10^−7^	0.13	0.48
7	cg02389743 *	chr2	152099098	*CACNB4*	Promoter	0.52	0.0006	0.92	2.08 × 10^−6^	−0.07	0.71
8	cg25771730	chr4	7425468	*SORCS2*	Intron	0.42	0.004	0.82	9.60 × 10^−6^	−0.17	0.38
9	cg00515982	chr5	181243930	*RACK1*	Promoter	−0.51	0.0004	−0.81	9.94 × 10^−6^	−0.02	0.91
10	cg09965760	chr6	151300350	*AKAP12*	Intron	0.44	0.0003	0.15	0.22	0.89	7.92 × 10^−6^

Illumina ID: CpG name in Illumina database; CHR: Chromosome; MAPINFO: Genomic coordinates; FC: fold change; *p*: *p*-value; * Novel discovery of DMPs.

**Table 3:** Summary of DMRs associated with MRONJ in the main analysis and subgroup analyses.

No	CHR	Gene Name	Main Analysis			First Subgroup Analysis			Second Subgroup Analysis		
			No. CpG	Mean Diff	*p*	No. CpG	Mean Diff	*p*	No. CpG	Mean Diff	*p*
1	chr6	*TNXB*	20	0.07	3.30 × 10^−10^	10	0.09	2.76 × 10^−7^	ND	ND	ND
2	chr17	*HOXB-AS3*, *HOXB3*, *HOXB4*	15	−0.06	4.27 × 10^−8^	ND	ND	ND	ND	ND	ND
3	chr4	*SPON2*	13	−0.03	2.76 × 10^−7^	12	−0.04	4.27 × 10^−8^	ND	ND	ND
4	chr6	*TRIM15*	9	0.07	1.43 × 10^−5^	12	0.1	3.30 × 10^−10^	ND	ND	ND

DMRs: Differentially methylated region; CHR: Chromosome; *p*: *p*-value; ND: Not detected; Mean Diff: Mean methylation difference.

**Table 4:** Correlation between DMPs and bone protein biomarkers.

No.	DMP	Methylation Status	Region	Main Analysis (*n* = 24)		First Subgroup Analysis (*n* = 15)		Second Subgroup Analysis (*n* = 9)		Biomarker
				r	*p*	r	*p*	r	*p*	
1	cg19137662 (*NOP56*)	Hypomethylation	Promoter	0.48	0.02	0.57	0.03	−0.40	0.28	OC
				0.32	0.13	0.38	0.16	0.73	0.025	RANKL
2	cg17230255 (*SCNN1A*)	Hypomethylation	Promoter	0.56	0.004	0.66	0.01	0.44	0.24	TNF- alpha
3	cg11392877 (*PDE8A*)	Hypomethylation	Promoter	−0.28	0.19	−0.51	0.05	0.63	0.07	SOST
				0.24	0.25	0.09	0.75	0.82	0.01	OPG

DMP: Differentially methylated probes.

## Data Availability

The original data may be made available upon reasonable request from the corresponding author, subject to privacy considerations.

## Supplementary Materials

The following supporting information can be downloaded at: https://www.mdpi.com/article/10.3390/ijms262211208/s1.
