# Supplementary material for: Integrative Epigenomic and Targeted Protein Analysis in MRONJ: Correlating DNA Methylation with Bone Biomarkers
Source: Int J Mol Sci. 2025 Nov 20;26(22):11208. doi: 10.3390/ijms262211208 (PMC12653879; doi:10.3390/ijms262211208)
Supplement: Supplementary file 1 [file ijms-26-11208-s001.zip › ijms-3908910-supplementary.pdf]

Supplemental materials

Article

Integrative Epigenomic and Targeted Protein Analysis in MRONJ: Correlating DNA Methylation with Bone Biomarkers

Raed Awadh Alshammari [1,2], Marwa Tantawy [1], Danxin Wang [1], Elysse Castro-Hall [3], Maria Abreu [3], Alessandro Villa [3], Joseph Katz [4], Lexie Shannon Holliday [5] and Yan Gong [1,6,*]

[1] Department of Pharmacotherapy and Translational Research and Center for Pharmacogenomics and Precision Medicine, College of Pharmacy, University of Florida, Gainesville, FL 32610, USA; raed.alshammari@ufl.edu (R.A.A.); mtantawy@cop.ufl.edu (M.T.)
[2] Department of Clinical Pharmacy, College of Pharmacy, University of Ha'il, Ha'il 55473, Saudi Arabia; rae.alshammari@uoh.edu.sa
[3] Miami Cancer Institute, Baptist Health South Florida, Miami, FL 33176, USA; elyssec@baptisthealth.net (L.H.); maria.abreu@baptisthealth.net (M.A.); alessandro.villa@baptisthealth.net (A.V.)
[4] Department of Oral Medicine, College of Dentistry, University of Florida, Gainesville, FL 32610, USA; jkatz@dental.ufl.edu
[5] Department of Orthodontics, College of Dentistry, University of Florida, Gainesville, FL 32610, USA; sholliday@dental.ufl.edu (L.S.H.)
[6] Cardio-Oncology Working Group, University of Florida Health Cancer Institute, Gainesville, FL 32610, USA
* Correspondence: gong@cop.ufl.edu; Tel.: +1-352-273-6297; Fax: +1-352-273-6121

Table S1. Clinical characteristics of study participants in the first subgroup analysis.

Variable	All Participants (*N*=15)		
	**Cases (*n*=7)**	**Control (*n*=8)**	**P value**
Continuous			
Age (years)	71.0 (63.0-74.0)	61.5 (52.5-66.8)	0.09
BMI (Kg/m^2)	29.75 (28.2-31.4)	26.4 (24.0-27.7)	0.23
Categorical			
Sex at birth			>0.99
Female	4 (57.1.%)	7 (87.5%)	
Male	3 (42.9%)	1 (12.5%)	
Race Groups			0.57
White	5 (71.4%)	7 (87.5%)	
Black	2 (28.6%)	1 (12.5%)	
Smoking status			>0.99
Current smoker	0 (0.0%)	1 (12.5%)	
Previous smoker	3 (42.9%)	4 (50.0%)	
Never smoke	4 (57.1%)	3 (37.5%)	
AAOMS Staging of MRONJ			
Stage 1	2 (28.6%)	-	
Stage2	3 (42.8%)	-	
Stage3	0 (0.0%)	-	
Stage4	0 (0.0%)	-	
NA	2 (28.6%)		
Lesion location			
Maxilla	0 (0.0%)	-	
Mandible	5 (71.4%)	-	
Anterior posterior	1 (14.3%)	-	
Lingual/buccal	0 (0.0%)	-	
NA	1 (14.3%)	-	

*For continuous variable, median and (IQR) were reported
Age was compared between groups using t-test, and BMI using Wilcoxon rank-sum test.

Abbreviations: MRONJ: medication-related osteonecrosis of the jaw; BMI: body mass index; AAOMS: American Association of Oral and Maxillofacial Surgeons; NA: Not Available.

Table S2. Clinical characteristics of study participants in the second subgroup analysis.

Variable	All Participants (*N*=9)		
	**Cases (*n*=5)**	**Control (*n*=4)**	**P value**
Continuous			
Age (years)	67.0 (52.0-70.0)	73.5 (71.5-75.5)	0.08
BMI (Kg/m^2)	25.7 (23.3-32.7)	28.9 (28.4-31.3)	0.23
Categorical			
Sex at birth			>0.99
Female	4 (80.0.%)	2 (50.0%)	
Male	1 (20.0%)	2 (50.0%)	
Race Groups			0.45
White	3 (60.0%)	4 (100.0%)	
Black	2 (40.0%)	0 (0.0%)	
Smoking status			>0.99
Current smoker	0 (0.0%)	0 (0.0%)	
Previous smoker	4 (80.0%)	3 (75.0%)	
Never smoke	1 (20.0%)	1 (25.0%)	
AAOMS Staging of MRONJ			
Stage 1	5 (100.0%)	-	
Stage2	0 (0.0%)	-	
Stage3	0 (0.0%)	-	
Stage4	0 (0.0%)	-	
Lesion location			
Maxilla	2 (40.0%)	-	
Mandible	2 (40.0%)	-	
Anterior posterior	0 (14.3%)	-	
Lingual/buccal	1 (20.0%)	-	

*For continuous variable, median and (IQR) were reported

Age was compared between groups using t-test, and BMI using Wilcoxon rank-sum test.

Abbreviations: MRONJ: medication-related osteonecrosis of the jaw; BMI: body mass index; AAOMS: American Association of Oral and Maxillofacial Surgeons; NA: Not Available

Table S3. The top DMPs identified in the main analysis of the MRONJ

No	Illumina ID	CHR	MAPINFO	Gene Name	CpG island	Feature	logFC	P
1	cg19137662*	chr20	2652716	NOP56	Island	Promoter	-1.38	2.19E-07
2	cg17230255*	chr12	6364225	SCNN1A	Shore	Promoter	-0.6	1.10E-06
3	cg27297662	chr21	31136999	TIAM1	Opensea	Intron	-1.3	2.89E-06
4	cg01876490	chr18	65766883	CDH7	Opensea	Intron	-1.1	5.10E-06
5	cg07863927	chr20	38643778	ARHGAP40	Shelf	Exon	0.52	5.80E-06
6	cg21289669	chr6	32096987	TNXB	Island	Exon	0.84	6.31E-06
7	cg16019932	chr12	31574769	DENND5B	Opensea	Intron	-0.57	7.90E-06
8	cg08869466	chr10	132929362	CFAP46	Shore	3′UTR	0.45	8.71E-06
9	cg20444539	chr1	193635643	ENSG00000227240	Opensea	intron	0.44	9.22E-06
10	cg17600926	chr8	52753918		Opensea	Intergenic	0.57	9.58E-06

Illumina ID: CpG name in Illumina database; CHR: Chromosome; MAPINFO: Genomic coordinates; FC: fold change; P: P-value; *Novel discovery of DMPs.

Table S4. The top DMPs identified in the first subgroup analysis of the MRONJ

No	Illumina ID	CHR	MAPINFO	Gene Name	CpG island	Feature	logFC	P
1	cg11392877	chr15	84981545	PDE8A	Island	Promoter	-0.98	5.35E-07
2	cg23786209	chr10	114525795	ABLIM1	Opensea	Intron	-1.04	1.45E-06
3	cg02389743*	chr2	152099098	CACNB4	Island	Promoter	0.92	2.08E-06
4	cg05561386	chr17	78459020	DNAH17	Island	Promoter	1.06	2.23E-06
5	cg04798497	chr3	156555236	SSR3	Island	Promoter	0.92	2.64E-06
6	cg01250643	chr19	6482209	DENND1C	Opensea	Promoter	-0.83	3.41E-06
7	cg19137662*	chr20	2652716	NOP56	Island	Promoter	-1.61	4.80E-06
8	cg24958206*	chr9	98056517	NANS	Island	Promoter	-0.92	6.22E-06
9	cg27297662	chr21	31136999	TIAM1	Opensea	Intron	-1.53	9.51E-06
10	cg25771730	chr4	7425468	SORCS2	Opensea	Intron	0.82	9.60E-06
11	cg00515982	chr5	181243930	RACK1	Island	Promoter	-0.81	9.94E-06

Illumina ID: CpG name in Illumina database; CHR: Chromosome; MAPINFO: Genomic coordinates; FC: fold change; P: P-value; *Novel discovery of DMPs.

Table S5. The top DMPs identified in the second subgroup analysis of the MRONJ

No	Illumina ID	CHR	MAPINFO	Gene Name	CpG island	Feature	logFC	P
1	cg27297893*	chr2	3131305		Opensea	Intergenic	-1.03	9.70E-07
2	cg02952972	chr11	972247	AP2A2	Shelf	Exon	0.99	1.52E-06
3	cg11062466	chr8	57143317	LINC01606	Island	Intron	2.25	3.07E-06
4	cg24797368*	chr19	55087890	EPS8L1	Shore	Promoter	1.66	5.59E-06
5	cg21062556*	chr1	28505270		Opensea	Promoter	0.86	5.88E-06
6	cg00788177	chr6	158939729		Island	Intergenic	1.85	6.32E-06
7	cg10016788	chr19	55087661	EPS8L1	Shore	Promoter	1.56	7.43E-06
8	cg09965760	chr6	151300350	AKAP12	Opensea	Intron	0.89	7.92E-06

Illumina ID: CpG name in Illumina database; CHR: Chromosome; MAPINFO: Genomic coordinates; FC: fold change; P: P-value; *Novel discovery of DMPs.

Table S6. Top 20 DMRs in the main analysis of the MRONJ

No	CHR	Start	End	Gene Name	No.CpG	Mean Difference	P
1	chr6	32095108	32097065	TNXB	20	0.07	3.30E-10
2	chr17	48572948	48576966	HOXB-AS3, HOXB3, HOXB4	15	-0.06	4.27E-08
3	chr4	1207704	1209178	SPON2	13	-0.03	2.76E-07
4	chr11	842140	846310	TSPAN4, POLR2L	12	-0.04	8.41E-07
5	chr20	767789	768976	SLC52A3	9	0.08	1.34E-05
6	chr1	156291214	156292480	TMEM79, GLMP	9	-0.05	1.41E-05
7	chr6	30162332	30163442	TRIM15	9	0.07	1.43E-05
8	chr18	26133638	26134120	PSMA8	9	0.06	1.45E-05
9	chr4	80196699	80198319	PRDM8, AC021127.1	9	-0.08	1.50E-05
10	chr17	48579181	48580895	HOXB-AS3, HOXB3, AC103702.1, MIR10A	9	-0.06	1.68E-05
11	chr3	184319342	184320529	EIF4G1	8	-0.06	3.46E-05
12	chr10	99535169	99537885	NKX2-3	8	-0.05	3.61E-05
13	chr6	1593831	1595506		8	0.07	3.65E-05
14	chr14	69849904	69850865		8	0.06	3.68E-05
15	chr16	67247429	67248934	SLC9A5, FHOD1	8	-0.01	3.70E-05
16	chr4	154781430	154782018	RBM46, AC009567.1	8	0.06	3.70E-05
17	chr10	122879460	122880266	C10orf88B, AC073585.1, FAM24B	8	0.07	3.72E-05
18	chr11	10693628	10694617	IRAG1	8	-0.02	3.72E-05
19	chr8	123181444	123182976	FAM83A, AC068228.1	8	0.06	3.73E-05
20	chr9	113056577	113057084	ZFP37	8	-0.07	3.76E-05

DMRs: Differentially methylated regions; CHR: Chromosome; P: P-value

Table S7. Top 20 DMRs in the first subgroup analysis of the MRONJ

No	CHR	Start	End	Gene Name	No.CpG	Mean Difference	P
1	chr6	30163042	30164938	TRIM15	12	0.1	3.30E-10
2	chr4	1207800	1209178	SPON2	12	-0.04	4.27E-08
3	chr6	32096214	32097008	TNXB	10	0.09	2.76E-07
4	chr6	33429345	33430900	SYNGAP1	9	-0.05	8.41E-07
5	chr20	767789	768976	SLC52A3	9	0.1	1.34E-05
6	chr17	7673474	7676007	TP53	9	-0.01	1.41E-05
7	chr11	4608127	4608437	TRIM68	8	0.09	1.43E-05
8	chr2	84516139	84516619	DNAH6	8	0.12	1.45E-05
9	chr6	31792606	31793278	VARS1	8	-0.07	1.50E-05
10	chr20	37518795	37519873	BLCAP	8	0.09	1.68E-05
11	chr9	122226773	122227731	LHX6	8	0.12	3.46E-05
12	chr19	57935301	57935592	ZNF418	7	-0.09	3.61E-05
13	chr11	1751238	1753523	AC068580.4, CTSD	7	-0.06	3.65E-05
14	chr20	64056973	64057595	TCEA2, AL355803.1	7	-0.08	3.68E-05
15	chr15	90929861	90930890	UNC45A, HDDC3	7	0.08	3.70E-05
16	chr6	151325165	151325682	AKAP12	7	-0.08	3.70E-05
17	chr15	25438253	25439702	UBE3A	7	-0.02	3.72E-05
18	chr20	62815610	62816733	COL9A3	7	0.04	3.72E-05
19	chr4	56681181	56681927	HOPX	7	-0.08	3.73E-05
20	chr6	28921768	28924235	TRIM27	7	0.0013	3.76E-05

DMRs: Differentially methylated regions; CHR: Chromosome; P: P-value

Table S8. Top 20 DMRs in the second subgroup analysis of the MRONJ

No	CHR	Start	End	Gene Name	No.CpG	Mean Difference	P
1	chr19	55087414	55088235	EPS8L1	6	0.18	0.0003
2	chr5	191371	191513	LRRC14B	6	0.19	0.0004

DMRs: Differentially methylated regions; CHR: Chromosome; P: P-value

Table S9. Biomarker summary: overall, MRONJ, and Control.

Biomarker	Overall (n=24) Mean ± SD	MRONJ (n=12) Mean ± SD	Control (n=12) Mean ± SD	P-value
TNF-α	4.14 ± 1.58	3.74 ± 1.19	4.53 ± 1.86	0.23
OPG	607.35 ± 281.26	668.77 ± 351.33	545.93 ± 183.69	0.53
OPN	51765.91 ± 64139.99	61804.82 ± 84569.28	41726.99 ± 35071.70	0.66
SOST	3434.72 ± 1434.56	3946.50 ± 1373.85	2922.94 ± 1357.97	0.08
OC	20654.18 ± 22532.52	15055.88 ± 6505.19	26252.49 ± 30836.46	0.36
RANKL	101.49 ± 106.76	76.99 ± 84.64	125.99 ± 123.92	0.34

Table S10. Cell Composition Summary – CP Method

Cell Composition Summary – CP Method

Cell.Type	MRONJ	Control	P-value
B	0.08 (0.06–0.08)	0.08 (0.06–0.11)	0.12
NK	0.11 (0.08–0.13)	0.12 (0.1–0.21)	0.22
CD4T	0.11 (0.07–0.12)	0.07 (0.04–0.15)	0.94
CD8T	0 (0–0)	0 (0–0)	0.22
Mono	0.11 (0.1–0.13)	0.1 (0.08–0.11)	0.40
Neutro	0.41 (0.35–0.46)	0.41 (0.31–0.46)	0.40
Eosino	0 (0–0)	0 (0–0)	0.19

Median and interquartile range (IQR) are reported.

CP, Constrained Projection (CP)

Table S11. Cell Composition Summary – CPS Method

Cell Composition Summary – CPS Method

Cell.Type	MRONJ	Control	P-value
B	0.02 (0–0.03)	0.02 (0.01–0.04)	0.17
NK	0.06 (0.03–0.09)	0.07 (0.04–0.15)	0.26
CD4T	0.07 (0.01–0.11)	0.03 (0.02–0.06)	0.27
CD8T	0.03 (0.01–0.07)	0.04 (0.01–0.15)	0.35
Mono	0.13 (0.1–0.15)	0.12 (0.09–0.13)	0.77
Neutro	0.66 (0.61–0.73)	0.64 (0.53–0.73)	0.34
Eosino	0 (0–0)	0 (0–0)	0.34

Median and interquartile range (IQR) are reported.

CPS, CIBERSORT-style Projection (CPS)

Table S12. Cell Composition Summary – RPC Method

Cell Composition Summary – RPC Method

Cell.Type	MRONJ	Control	P-value
B	0.01 (0–0.02)	0.01 (0–0.04)	0.12
NK	0.06 (0.03–0.08)	0.07 (0.05–0.15)	0.19
CD4T	0.06 (0.02–0.13)	0.03 (0.02–0.07)	0.36
CD8T	0.06 (0–0.08)	0.05 (0.01–0.15)	0.47
Mono	0.12 (0.09–0.14)	0.11 (0.08–0.11)	0.63
Neutro	0.66 (0.59–0.72)	0.65 (0.5–0.73)	0.36
Eosino	0 (0–0)	0 (0–0)	NA

Median and interquartile range (IQR) are reported.

RPC,Robust Partial Correlations (RPC)

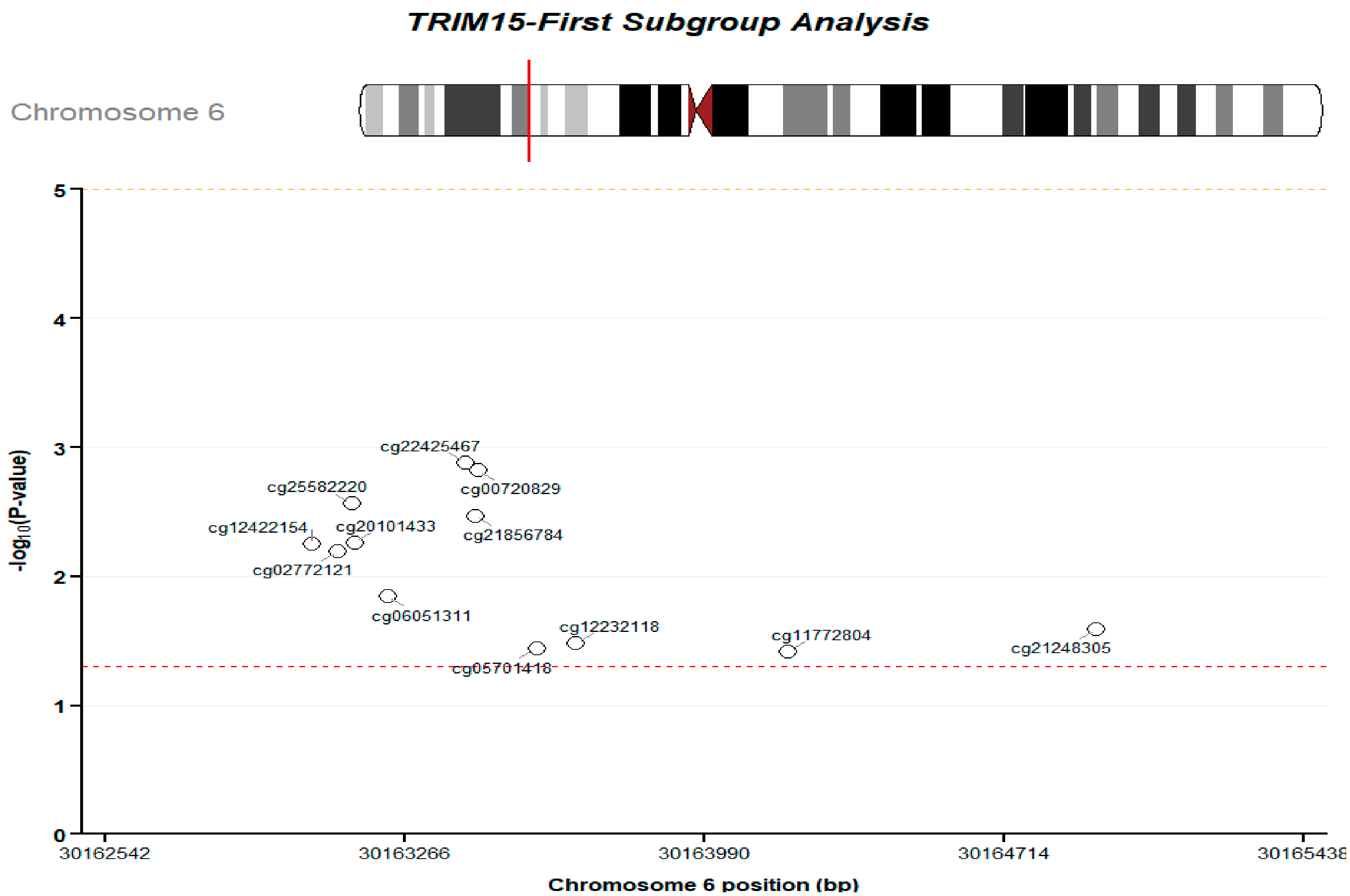

Figure S1. Locus plot of the *TRIM15* DMR, with each dot representing a CpG site annotated to *TRIM15*. The x-axis shows genomic coordinates, and the y-axis shows –log10(p-value) for the association of each individual CpG site in the first subgroup analysis.

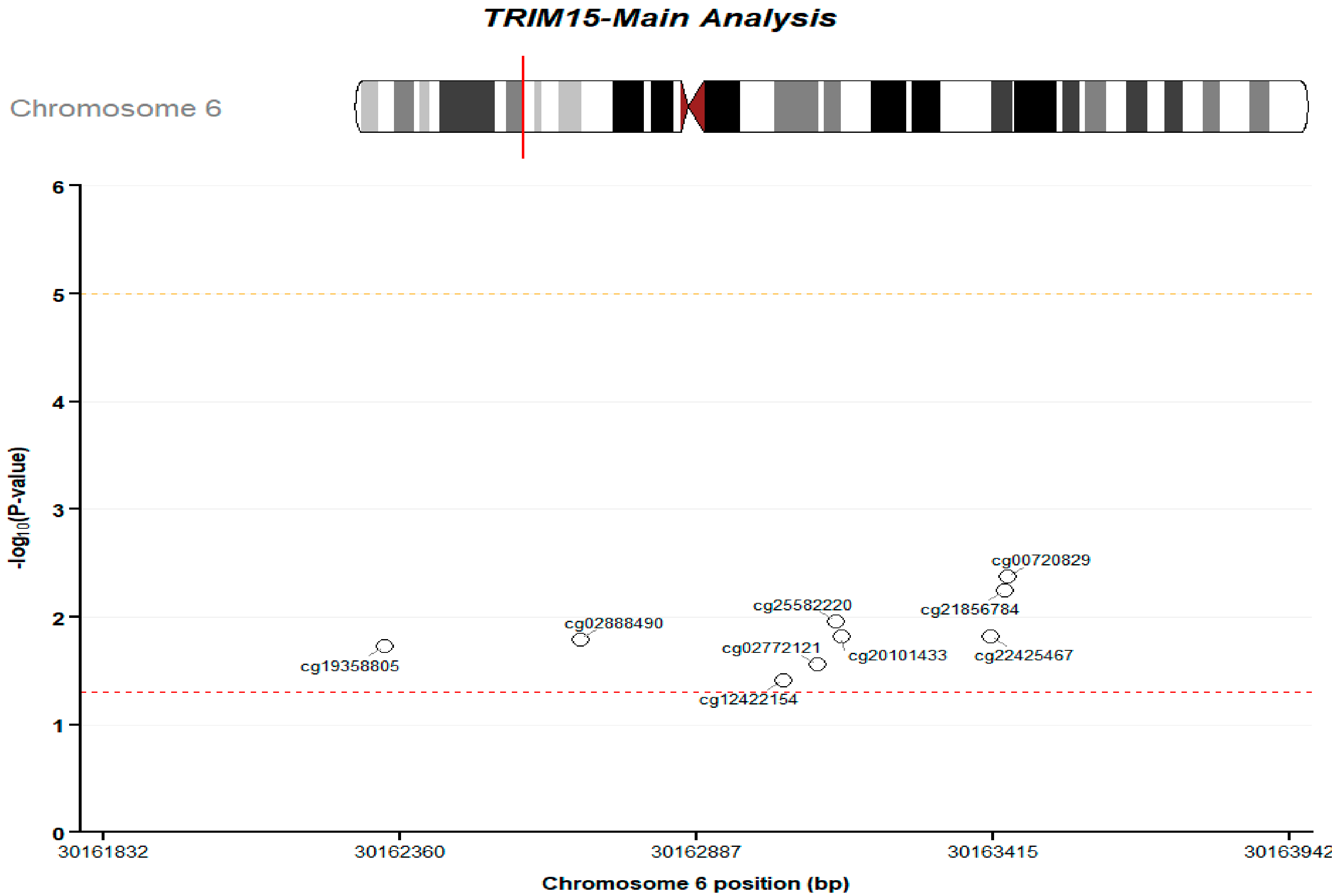

Figure S2. Locus plot of the *TRIM15* DMR, with each dot representing a CpG site annotated to *TRIM15*. The x-axis shows genomic coordinates, and the y-axis shows –log10(p-value) for the association of each individual CpG site in the main analysis.

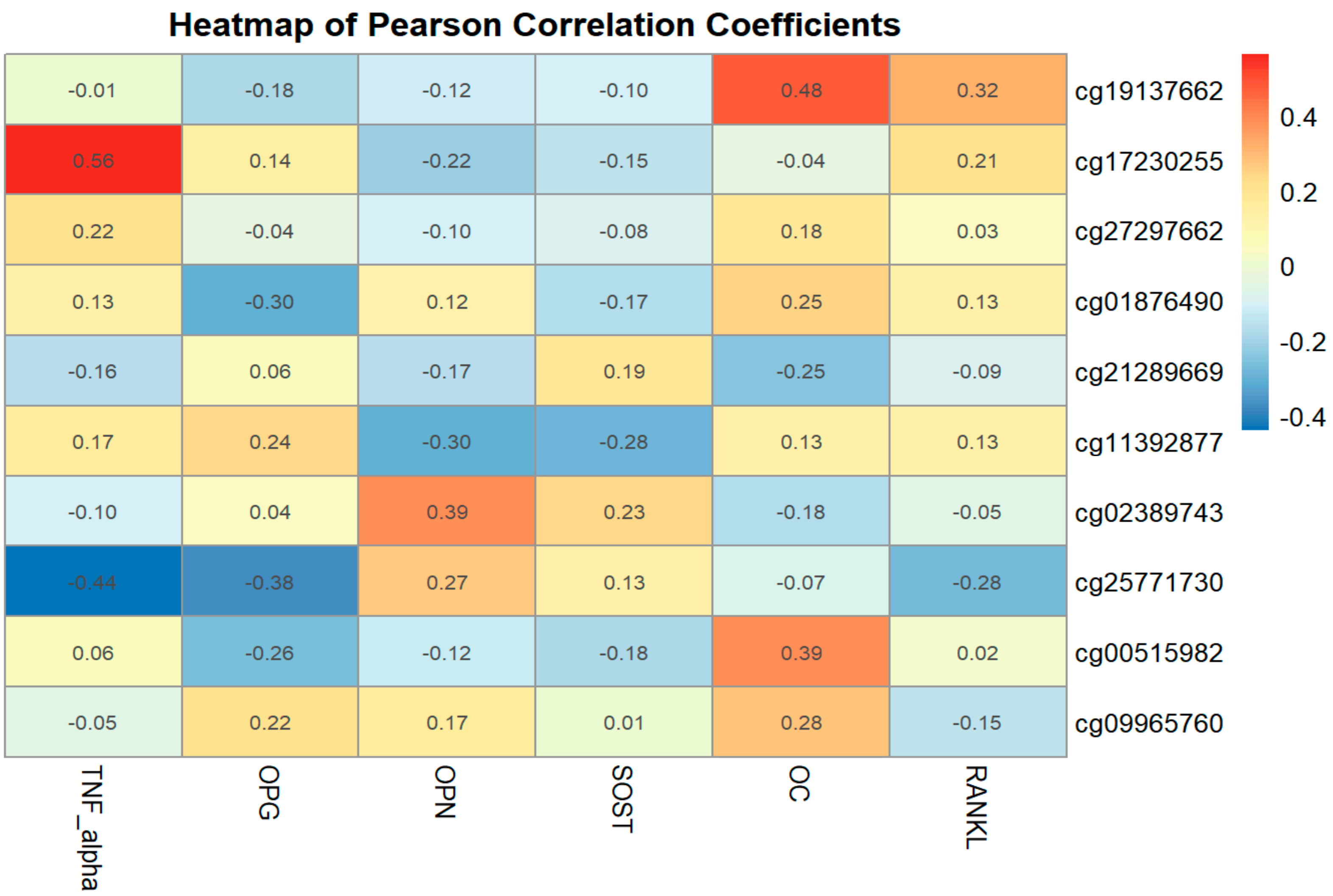

Figure S3. Heatmap of Pearson correlation showing correlation coefficients of bone protein biomarkers with selected DMPs in the main analysis

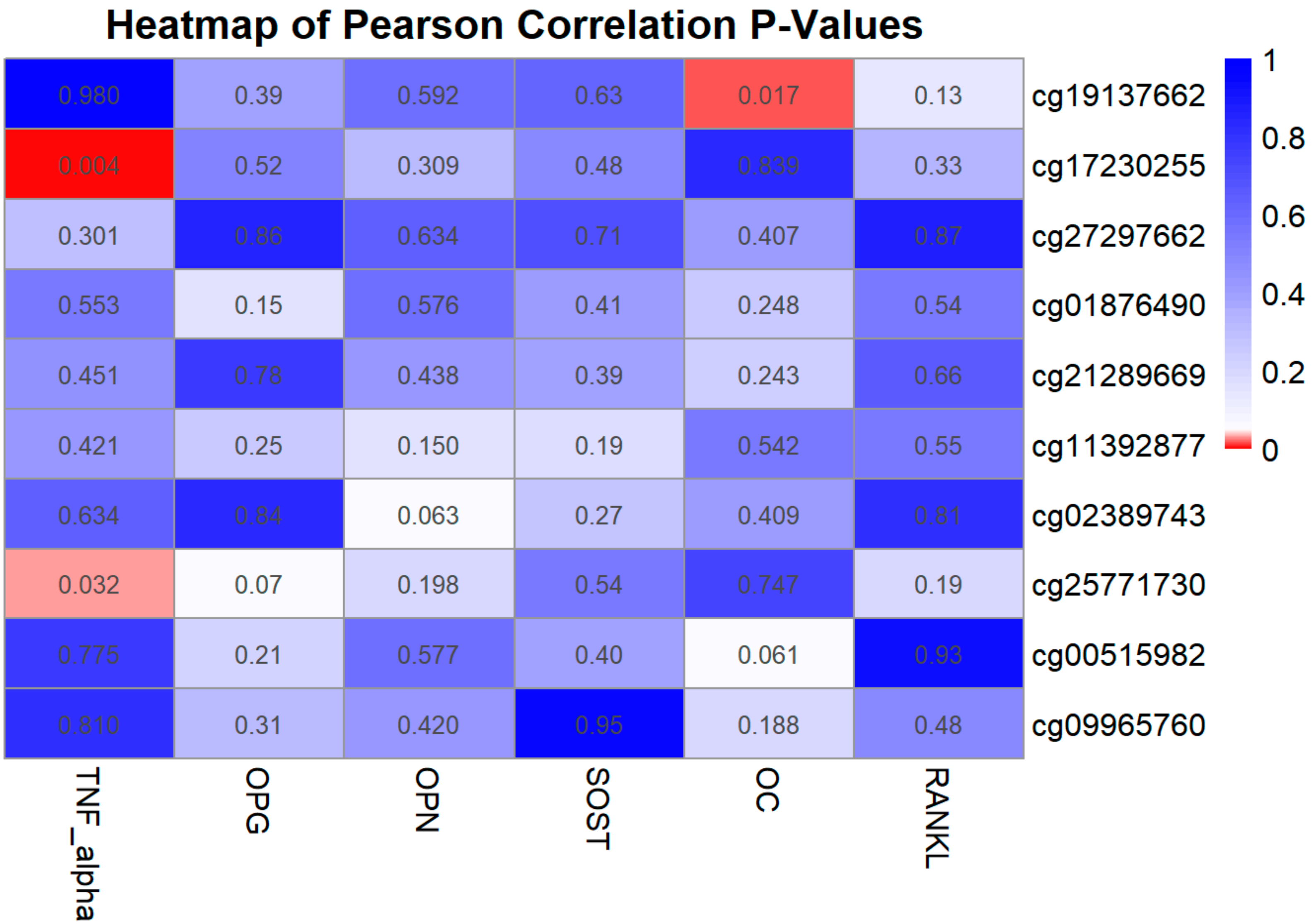

Figure S4. Heatmap of Pearson correlation showing p-values of bone protein biomarkers with selected DMPs in the main analysis

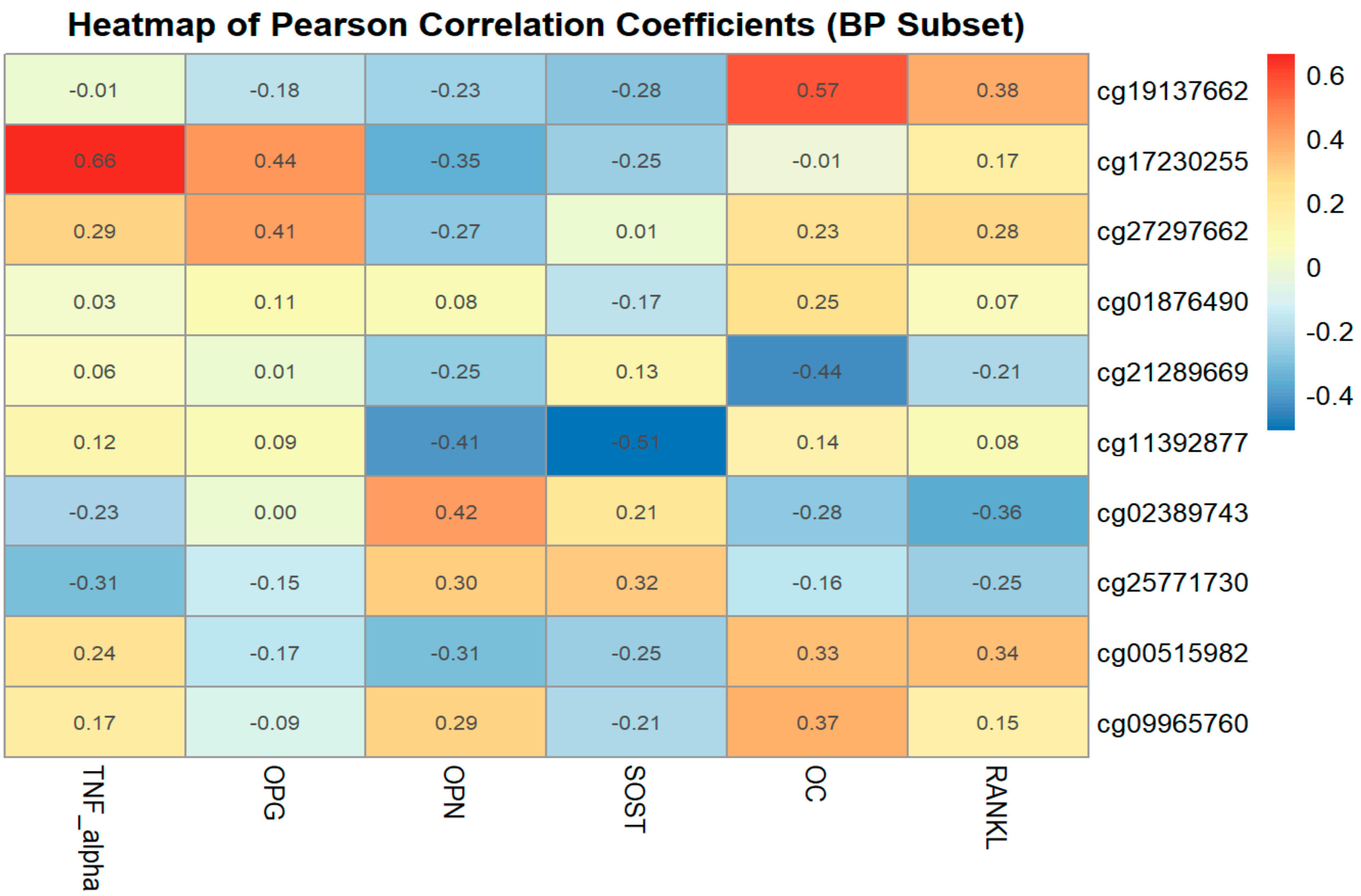

Figure S5. Heatmap of Pearson correlation showing correlation coefficients of bone protein biomarkers with selected DMPs in the first subgroup analysis

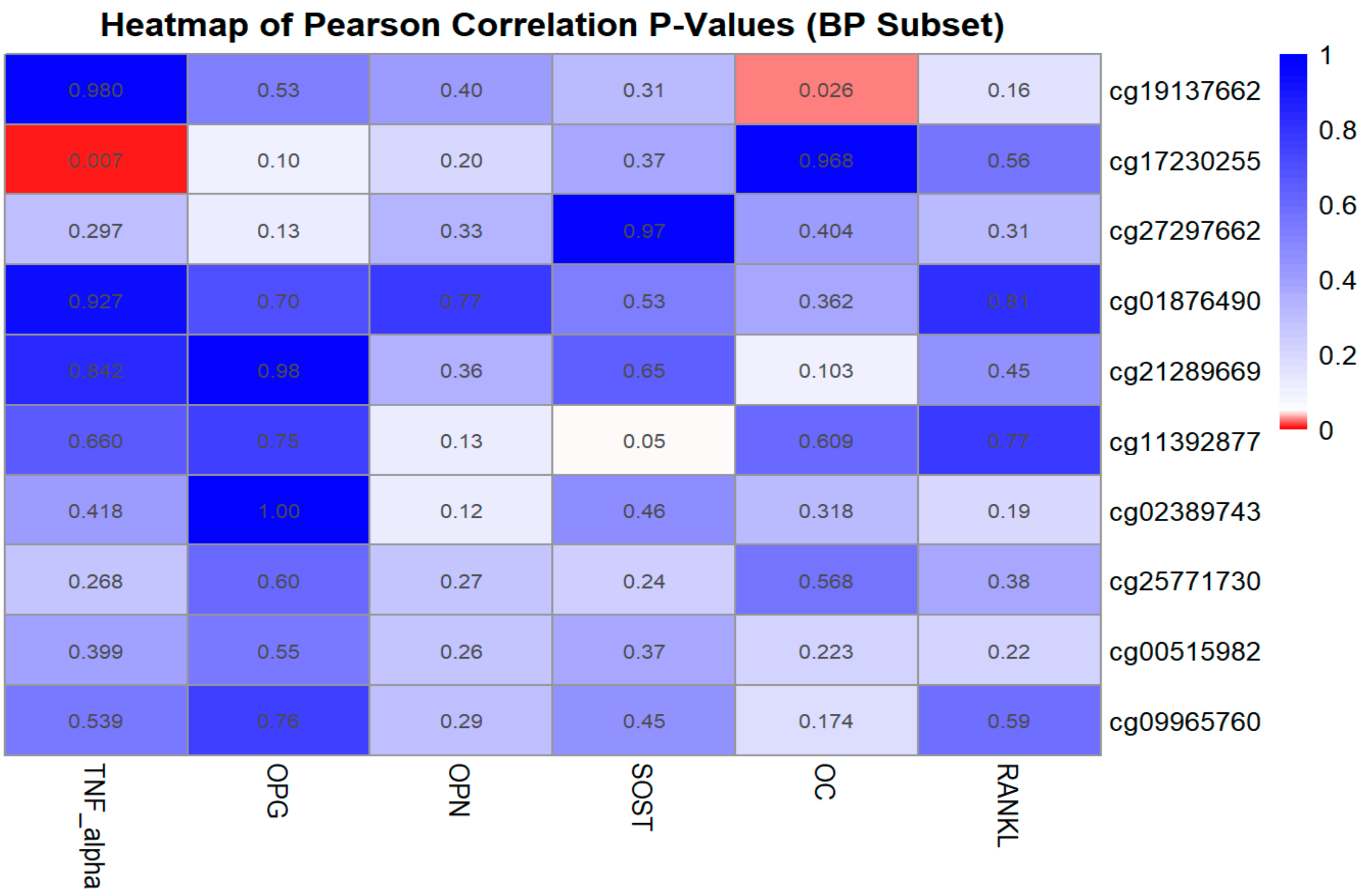

Figure S6. Heatmap of Pearson correlation showing p-values of bone protein biomarkers with selected DMPs in the first subgroup analysis

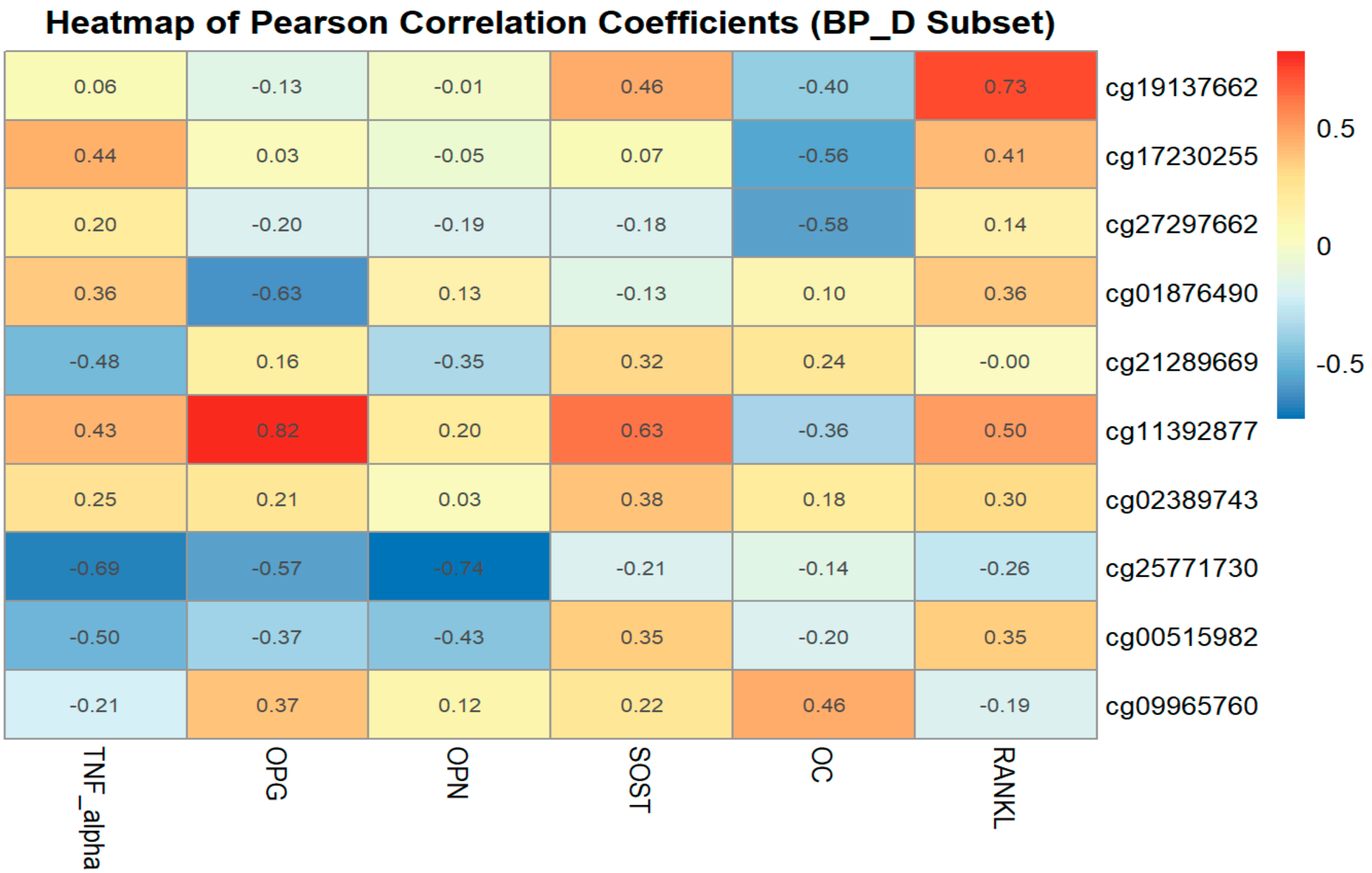

Figure S7. Heatmap of Pearson correlation showing correlation coefficients of bone protein biomarkers with selected DMPs in the second subgroup analysis

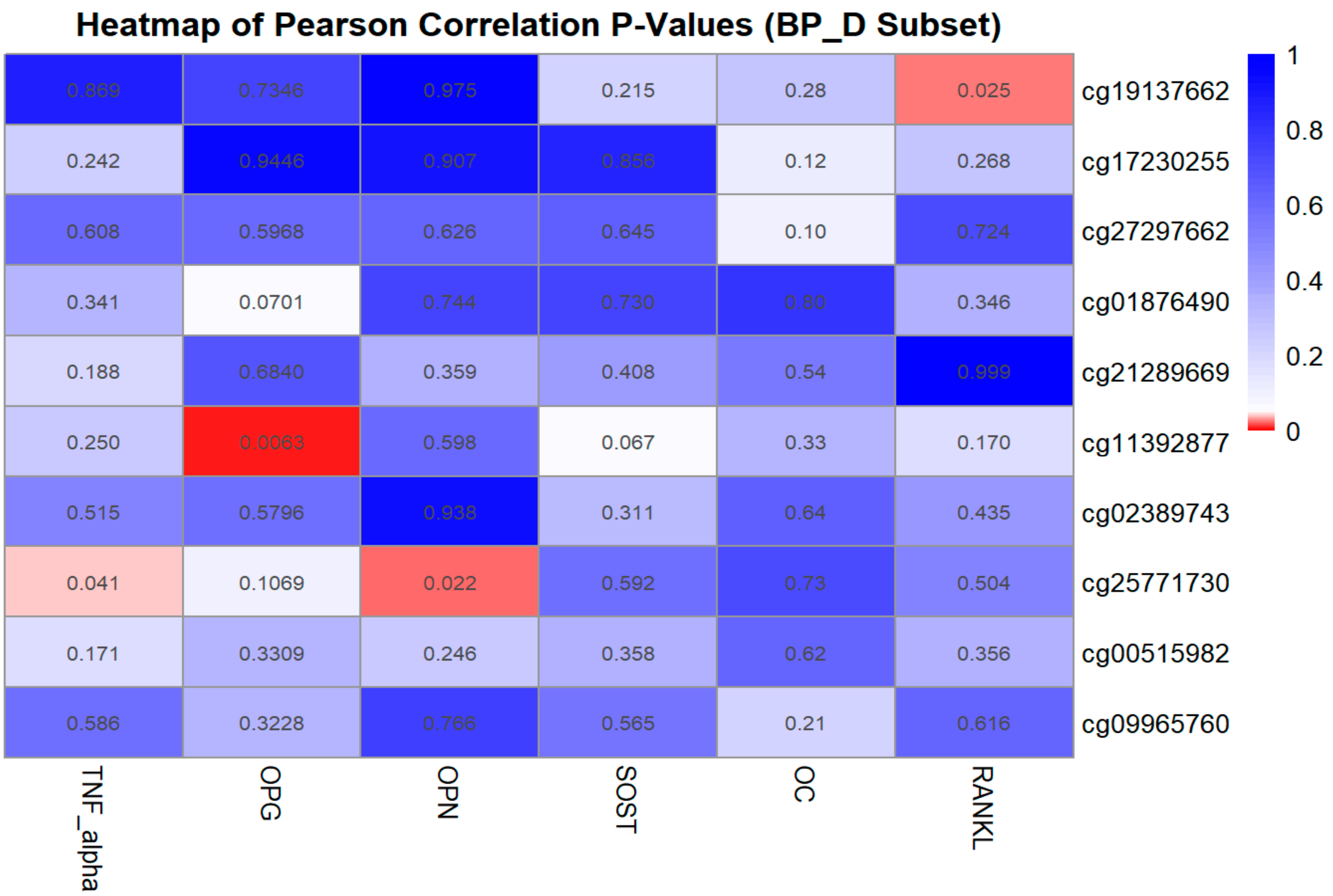

Figure S8. Heatmap of Pearson correlation showing p-value of boen protein biomarkers with selected DMPs in the second subgroup analysis

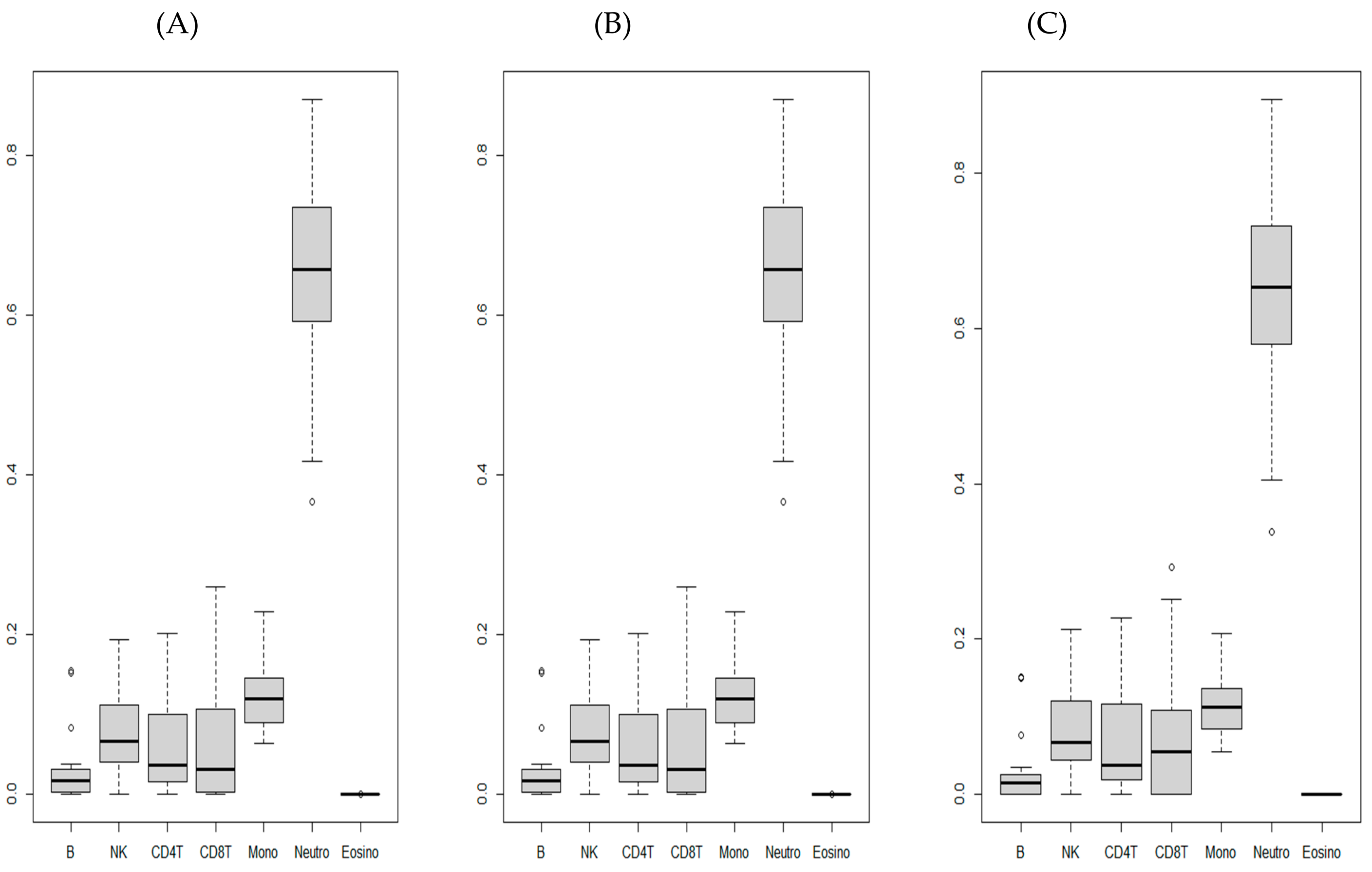

Figure S9. Estimating cell type proportions in the main analysis using three methods. (A); Constrained Projection (CP) also known as Houseman (B); Cibersort (CBS); (C); Robust Partial Correlations (RPC)
